# A Peptide of SPARC Interferes with the Interaction between Caspase8 and Bcl2 to Resensitize Chemoresistant Tumors and Enhance Their Regression In Vivo

**DOI:** 10.1371/journal.pone.0026390

**Published:** 2011-11-01

**Authors:** Mahbuba Rahman, Annie P. K. Chan, Isabella T. Tai

**Affiliations:** 1 Division of Gastroenterology, University of British Columbia, Vancouver, British Columbia, Canada; 2 Genome Sciences Centre, British Columbia Cancer Agency, Vancouver, British Columbia, Canada; National Taiwan University Hospital, Taiwan

## Abstract

SPARC, a matricellular protein with tumor suppressor properties in certain human cancers, was initially identified in a genome-wide analysis of differentially expressed genes in chemotherapy resistance. Its exciting new role as a potential chemosensitizer arises from its ability to augment the apoptotic cascade, although the exact mechanisms are unclear. This study further examines the mechanism by which SPARC may be promoting apoptosis and identifies a smaller peptide analogue with greater chemosensitizing and tumor-regressing properties than the native protein. We examined the possibility that the apoptosis-enhancing activity of SPARC could reside within one of its three biological domains (N-terminus (NT), the follistatin-like (FS), or extracellular (EC) domains), and identified the N-terminus as the region with its chemosensitizing properties. These results were not only confirmed by studies utilizing stable cell lines overexpressing the different domains of SPARC, but as well, with a synthetic 51-aa peptide spanning the NT-domain. It revealed that the NT-domain induced a significantly greater reduction in cell viability than SPARC, and that it enhanced the apoptotic cascade via its activation of caspase 8. Moreover, in chemotherapy resistant human colon, breast and pancreatic cancer cells, its chemosensitizing properties also depended on its ability to dissociate Bcl2 from caspase 8. These observations translated to clinically significant findings in that, in-vivo, mouse tumor xenografts overexpressing the NT-domain of SPARC had significantly greater sensitivity to chemotherapy and tumor regression, even when compared to the highly-sensitive SPARC-overexpressing tumors. Our results identified an interplay between the NT-domain, Bcl2 and caspase 8 that helps augment apoptosis and as a consequence, a tumor's response to therapy. This NT-domain of SPARC and its 51-aa peptide are highly efficacious in modulating and enhancing apoptosis, thereby conferring greater chemosensitivity to resistant tumors. Our findings provide additional insight into mechanisms involved in chemotherapy resistance and a potential novel therapeutic that specifically targets this devastating phenomenon.

## Introduction

**Figure 1:**
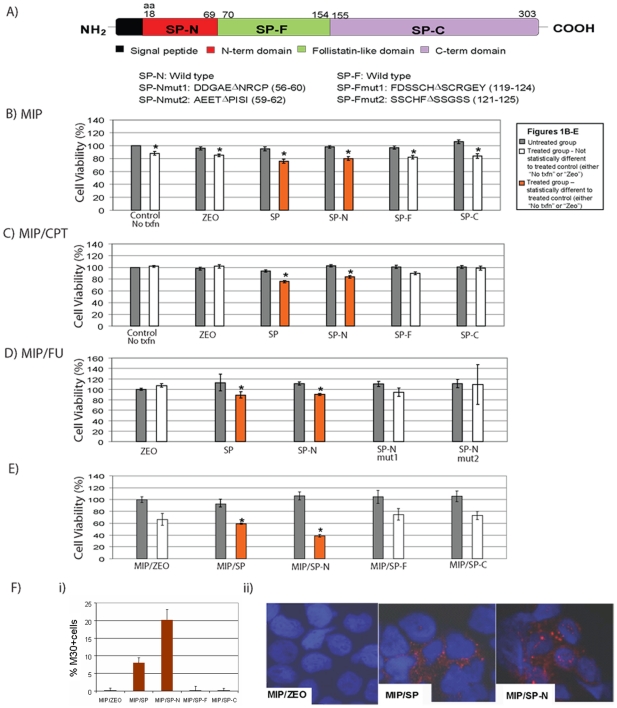
Over-expression of the N-terminus domain of SPARC diminished cell viability and induced apoptosis in colorectal cancer cell lines. **A**) The biological domains of SPARC (SP) and sites of mutations introduced within the N-terminus (SP-N), follistatin-like (SP-F), and extracellular (SP-C) domains are represented. **B-D**) The effect on cell viability following over-expression of SPARC domains were assessed by transient transfection of sensitive (**B**) and (**C**) CPT resistant MIP101 cells (MIP/CPT) with plasmid constructs for SP, SP-N, SP-F, SP-C, and empty vector (ZEO) for 48 h, followed by no treatment (gray bars) or treatment (white or red bars) with 100 µM CPT-11, for an additional 24 h. Cell viability was assessed by WST assay. Transfection with the empty vector (ZEO) served as control for all experiments; “No txfn” = untransfected cells. **D**) Transfection of chemoresistant MIP/5FU cells with different mutations introduced into SP-N (SP-N mut 1 or SP-N mut2) no longer diminished the survival of cells exposed to 1000 µM 5-FU for 24 hrs. **E**) Cell lines stably transfected with and overexpressing SPARC (MIP/SP), SP-N (MIP/SP-N), SP-F (MIP/SP-F), SP-C (MIP/SP-C), and empty-vector (MIP/ZEO) were treated with 5 µM 5-FU for 24 h. (*) = statistically different (*p*<0.05) from respective untreated control (B–E) **F**) Stable MIP101 cells overexpressing SPARC, the different SPARC domains or empty-vector control were assessed for apoptosis by M30 staining: i) Comparison of % M30 positive cells in different cell lines after exposure to 5 µM 5-FU for 24 h, ii) representative images from M30 staining in cells after 5-FU treatment. Results represent mean ± s.e. (n = 3–4 independent studies).

Many pathological conditions arise because of abnormal regulation in cellular activities, such as apoptosis, that disrupt the fine balance between cell survival and death. This dysregulation can contribute to cancer initiation, progression, and even influence a tumor's response to chemotherapy. SPARC (secreted protein and rich in cysteine), a matricellular protein found to be underexpressed in certain cancers, has emerged as a multifunctional protein capable of inhibiting the growth of neuroblastomas [1], leukemia [2], pancreatic [3], colorectal [4] and ovarian cancers [5]. Its pro-apoptotic activity in ovarian, pancreatic, lung and colorectal cancers (CRC) [4], [6], [7], is also thought to enhance chemotherapeutic response and reverse drug resistance [4], [8]. Recent studies revealed that the recruitment and propagation of the apoptotic cascade involved the interaction between the N-terminus of caspase 8 and SPARC [8]. In this study, the mechanisms involved in SPARC-mediated apoptosis are further examined, with a specific focus on identifying a region within SPARC that may be responsible for promoting apoptosis. This is based on reports that the three structural domains of SPARC contribute to this protein's multi-functional yet distinct biological properties (Fig. 1A): (1) N-terminus (NT), (2) follistatin-like (FS), and (3) the extracellular C-terminus (EC) domains [9], [10]. For example, the N-terminus contributes to its cell spreading properties [11], the follistatin-like domain contains cysteine-rich residues, and has been shown to inhibit endolethial cell migration [12], [13], while the C-terminus contains the extracellular Ca^2+^-binding module [14] and may have anti-angiogenic properties [11], [13], [15].

Our current study demonstrates that the pro-apoptotic activity of SPARC is confined to a specific region of the protein, and that a recombinant peptide containing this smaller region alone is capable of conferring greater apoptosis and tumor regression in vivo. In addition, while we previously demonstrated an interaction between SPARC and caspase 8 in potentiating the apoptotic cascade [8], this study invokes Bcl2, an anti-apoptotic member of the intrinsic/mitochondrial pathway of apoptosis, as an important component in this interaction with caspase 8 and SPARC. This network of interactions affects the apoptotic cascade which then influences drug sensitivity, therapy response and reversal of drug resistance.

## Results

### Effect of different SPARC domains on apoptosis

We previously showed that exposure to high levels of SPARC enhances apoptosis and significantly reduces cell viability in CRC cells that have become resistant to chemotherapy [4], [8]. Although a number of biological activities (such as inhibition of angiogenesis and proliferation) have already been ascribed to smaller proteolytic cleavage products of SPARC [10], it is not known if any of them also induce apoptosis similar to the native protein. Therefore, in order to investigate this possibility, sensitive and resistant CRC MIP101 cells were transiently transfected with vectors containing only the N-terminus (SP-N), the follistatin-like (SP-F), or the C-terminus (SP-C) domains of SPARC, or mutant domains (SP-Nmut1, SP-Nmut2, SP-Fmut1, SP-Fmut2) (Fig. 1A). In the chemotherapy naïve (MIP101) and resistant cells (to CPT-11, MIP/CPT; or to 5-FU, MIP/5FU) examined, transient over-expression of SP-N reduced cell viability (Fig. 1B–D) in response to chemotherapy. In sensitive cells, such as MIP101, this represented an additional decrease in viability of 37.18±3.65% (p<0.005) following transfection with SP-N (Fig. 1B), in comparison to 5-FU-treated ZEO (empty vector)-transfected cells. Even more significant is the observation that SP-N also decreased cell viability in the chemo-resistant cells examined (Fig. 1C, D).

Based on these initial results suggesting that a recombinant protein containing only the N-terminus domain of SPARC was capable of diminishing cell viability in not only sensitive CRC cells, but also in their chemo-resistant counterparts, we decided to validate this observation by mutating the N-terminus domain, and found that the mutant forms no longer promoted a chemosensitizing effect. In resistant MIP/5FU cells, transient over-expression of two different mutants of SP-N (SP-Nmut1, SP-Nmut2) failed to increase their sensitivity to 5-FU (Fig. 1D), while over-expression of wild-type SP-N was able to decrease cell viability by 24.5±0.8% (p<0.005) in response to 5-FU.

This negative effect of the N-terminus domain of SPARC on cell viability led us to further evaluate them using stable clones of MIP101 cells over-expressing the N-terminus (MIP/SP-N), the follistatin-like (MIP/SP-F), or the extracellular (MIP/SP-C) domains. MIP/SP and MIP/SP-N cells showed the greatest decrease in cell viability and proliferation after 5-FU treatment, suggesting enhanced chemosensitivity in comparison to control MIP/ZEO cells (Fig. 1E). This reduction in cell viability resulting from overexpression of the N-terminus domain of SPARC was associated with a 20-fold increase in the percentage of MIP/SP-N cells undergoing apoptosis in response to 5-FU (Fig. 1F). A similar but less dramatic response is seen with MIP/SP cells (Fig. 1F), while no significant increase in apoptosis were observed in MIP/SP-F or MIP/SP-C following exposure to 5-FU.

### N-terminus domain of SPARC and apoptosis

**Figure 2:**
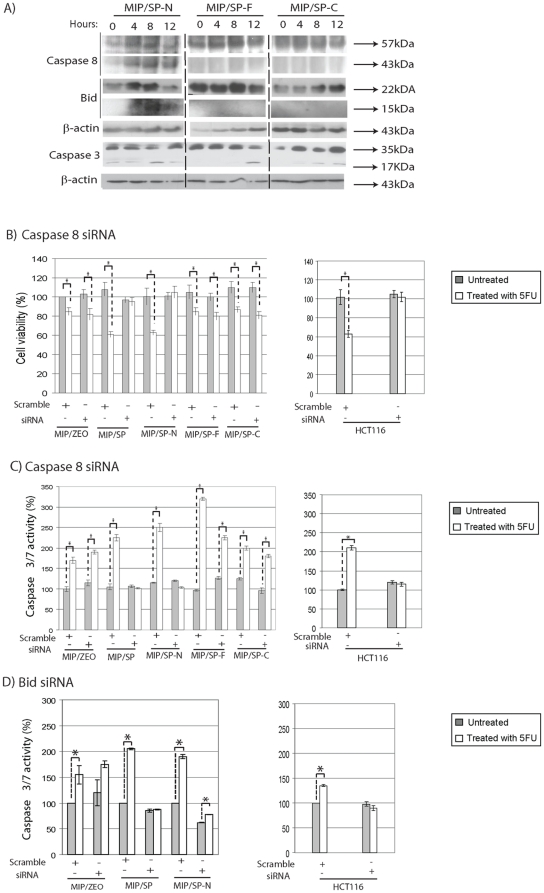
N-terminus domain of SPARC activates caspase 8 in response to treatment with 5-FU. **A**) Changes in the expression of proteins involved in the apoptotic pathway following incubation with 5-FU at different time points in MIP101 cells stably overexpressing SPARC domains by immunoblotting. **B–D**) The contribution of caspase 8 or Bid in inducing SP-N-associated apoptosis were compared with various cell lines after transient transfection with caspase 8 and Bid siRNA (or scramble control) for 48 h, followed by no treatment (grey bars) or incubation with 5-FU (white or orange bars) for 24 h. Cell viability after caspase 8 siRNA transfection was assessed by WST assay (**B**), while apoptosis after caspase 8 (**C**) or Bid (**D**) siRNA transfection was assessed by caspase 3/7 assay. Results represent mean ± s.e. (n = 3–4 independent studies). *p<0.05, Student's t-test, in comparison to untreated controls.

We previously reported that SPARC-mediated apoptosis involved the activation of the extrinsic pathway, via caspase 8 [8]. Given the above results, the possibility that the N-terminus domain of SPARC may engage in a similar mechanism to enhance apoptosis was investigated. Following exposure to 5-FU, caspase 8 activity (based on its cleaved product, 43 kDa) was mainly observed in MIP/SP-N cells (Fig. 2A). There was also higher expression of cleaved-BID and earlier activation of caspase 3 in MIP/SP-N cells (by 8 hrs after exposure to 5-FU), suggesting a similar involvement of the extrinsic pathway in cells over-expressing the N-terminus domain. To further validate the involvement of caspase 8 and Bid in the N-terminus-mediated apoptotic events, siRNA knock-down studies were undertaken which revealed that the absence of caspase 8 eliminated the apoptotic response following treatment with chemotherapy in MIP/SP and MIP/SP-N cells, as measured by cell viability assays (Fig. 2B) and caspase 3/7 activity (Fig. 2C). Similarly, in the absence of Bid following siRNA knock-down, MIP/SP and MIP/SP-N cells also demonstrated a diminished caspase 3/7 response to chemotherapy (Fig. 2D). Interestingly, while intrinsically high SPARC-expressing HCT116 cells were also no longer responsive to chemotherapy following *caspase 8* and *Bid* gene-silencing, cells overexpressing the FS- and EC-domains of SPARC were unaffected by *caspase 8* or *Bid* knock-down (Fig. 2B–D).

### Tumor xenografts overexpressing the N-terminus domain of SPARC are more chemosensitive

The results of the in-vitro studies indicate that the N-terminus domain of SPARC alone is capable of enhancing chemosensitivity. This, together with previous in-vivo studies demonstrating greater tumor regression of xenografts of MIP/SP cells to chemotherapy [4] led us to examine if tumor xenografts overexpressing the N-terminus domain of SPARC also had a heightened sensitivity to chemotherapy.

**Figure 3:**
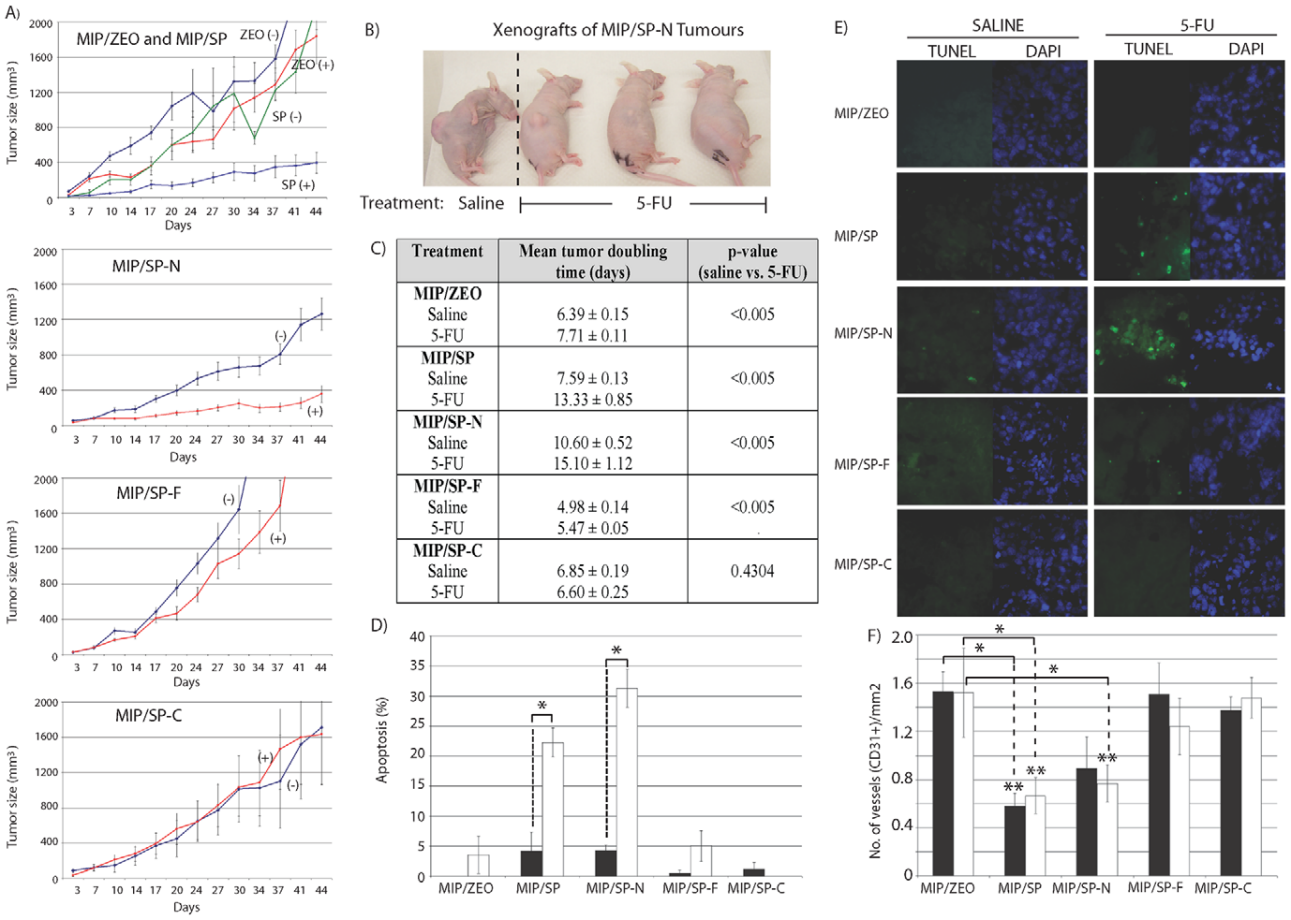
Xenografts overexpressing SP-N have greater tumor regression and apoptosis in response to 5-FU. **A,B**) Comparison of tumor size in xenografts from MIP/SP-N, MIP/SP-F, MIP/SP-C, and MIP/ZEO cells with or without treatment with 5-FU; “+” = 5-FU-treatment; “−” = saline-treatment, (n = 14/group; mean ± s.e). **C**) Tumor doubling times of xenografts following 5-FU treatment. (**D**) Percentage of apoptotic cells detected by TUNEL staining [representative images in (**E**)]. (**F**) number of CD31-positive stained blood vessels in the tumor xenografts of (▪) saline or (□) 5-FU-treated animals. Student's t-test * p<0.05, ** p<0.01.

Indeed, in-vivo, MIP/SP-N cells were also more sensitive to chemotherapy than xenografts of MIP/ZEO control cells: xenografts of tumors over-expressing either the N-terminus domain or SPARC were the most responsive to 5-FU treatment, as tumors remained <400 mm^3^ after 41 days of treatment, while saline-treated animals harbored tumors greater than 1144.1±181.3 mm^3^ (Fig. 3A,B). Tumor xenografts of cells overexpressing the other domains of SPARC did not differ in size between the treatment and control groups. There was also a significantly longer tumor doubling times for MIP/SP and MIP/SP-N saline-treated xenografts, in comparison to MIP/ZEO xenografts (p<0.005; Fig. 3C). Xenografts of MIP/SP-C had a similar tumor doubling time as MIP/ZEO (p = 0.07), while MIP/SP-F had a faster growth rate (p<0.005).

The extent of tumor regression correlated with a greater percentage of cells undergoing apoptosis in xenografts of MIP/SP (22.2±2.4% vs 4.2±3.1% in control, p = 0.01) and MIP/SP-N (31.3±3.2% vs 4.3±0.9% in control, p<0.005) cells following treatment with 5-FU in comparison to their saline-treated counterparts (Fig. 3D, E). Caspase 3 (cleaved) expression was also highest in tumors of MIP/SP-N and MIP/SP cells (Figure S1). No differences were detected in xenografts of the other cell lines, despite 5-FU treatment. Angiogenesis also appeared to be affected, as xenografts of MIP/SP and MIP/SP-N cells showed significantly fewer CD31+ staining than xenografts of MIP/ZEO, MIP/SP-F or MIP/SP-C (p<0.05, Fig. 3F). These findings demonstrate that a fragment of SPARC containing only its N-terminus domain has similar biological activities as the full-length protein in relation to its ability to promote apoptosis while inhibiting angiogenesis [4], [16].

### The N-terminus of SPARC interacts with caspase 8 and prevents its interaction with Bcl2

**Figure 4:**
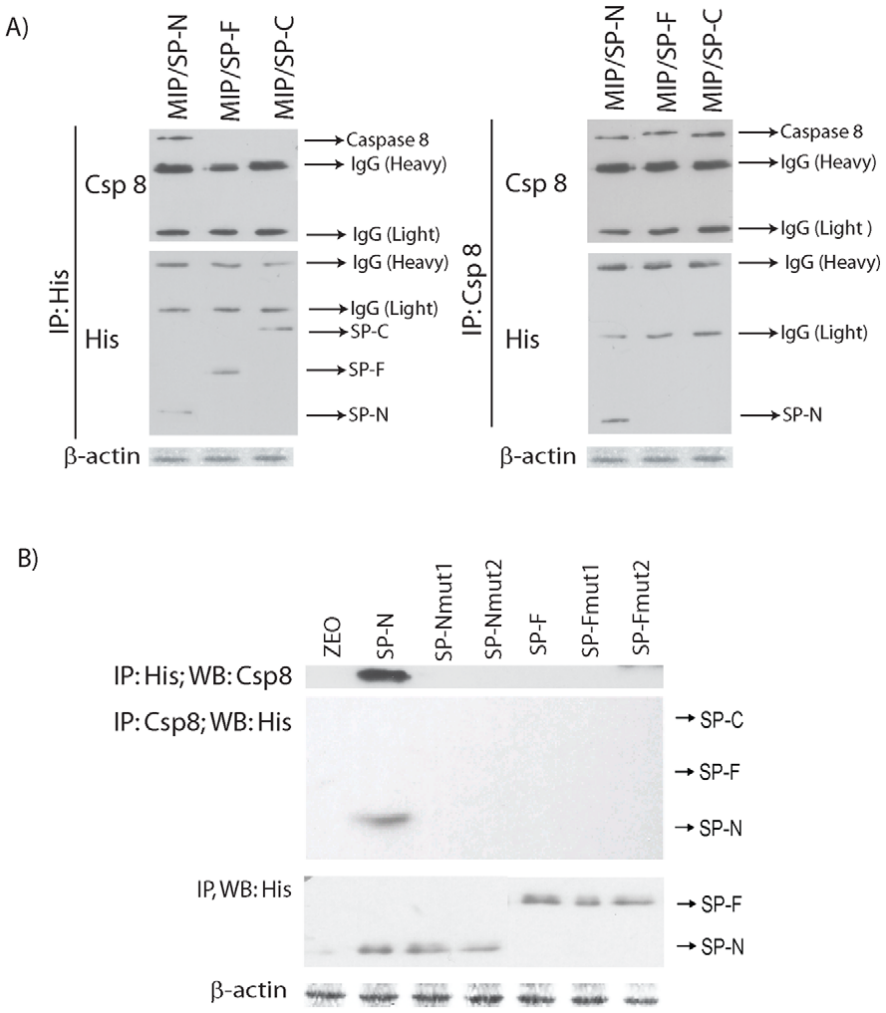
The site of interaction between SPARC and caspase 8 is at the N-terminus of SPARC. Co-localization of the N-terminus of SPARC with caspase 8 was determined by co-immunoprecipitation (IP) studies using whole protein extracts. **A**) Only the recombinant N-terminus containing His-tagged fusion protein from MIP/SP-N cells co-IP with caspase 8 in a reciprocal fashion. **B**) Mutations in the N-terminus domain of SPARC (SP-Nmut1, SP-Nmut2) abolishes this interaction with caspase 8, and only the wild-type SP-N interacted with caspase 8. As additional controls, neither the wild type nor the mutant FS domain (SP-Fmut1, SP-Fmut2) interacted with caspase 8.

The ability of SPARC to promote greater apoptosis in-vitro and in-vivo following exposure to cytotoxic agents [4] results, in part, from its interaction with caspase 8 leading to the activation of the extrinsic pathway of apoptosis [8]. A similar interaction was detected with the N-terminus domain of SPARC, but not the FS- or EC-domains, following co-immunoprecipitation studies using antibodies against caspase 8 and His_6_ (to detect fusion proteins of the NT, FS or EC-domains) (Fig. 4A). To further validate this observation, cells transiently overexpressing the wild-type NT-domain effectively co-immunoprecipitated caspase 8, while this interaction was absent with mutant proteins SP-Nmut1, and SP-Nmut2 (Fig. 4B).

**Figure 5:**
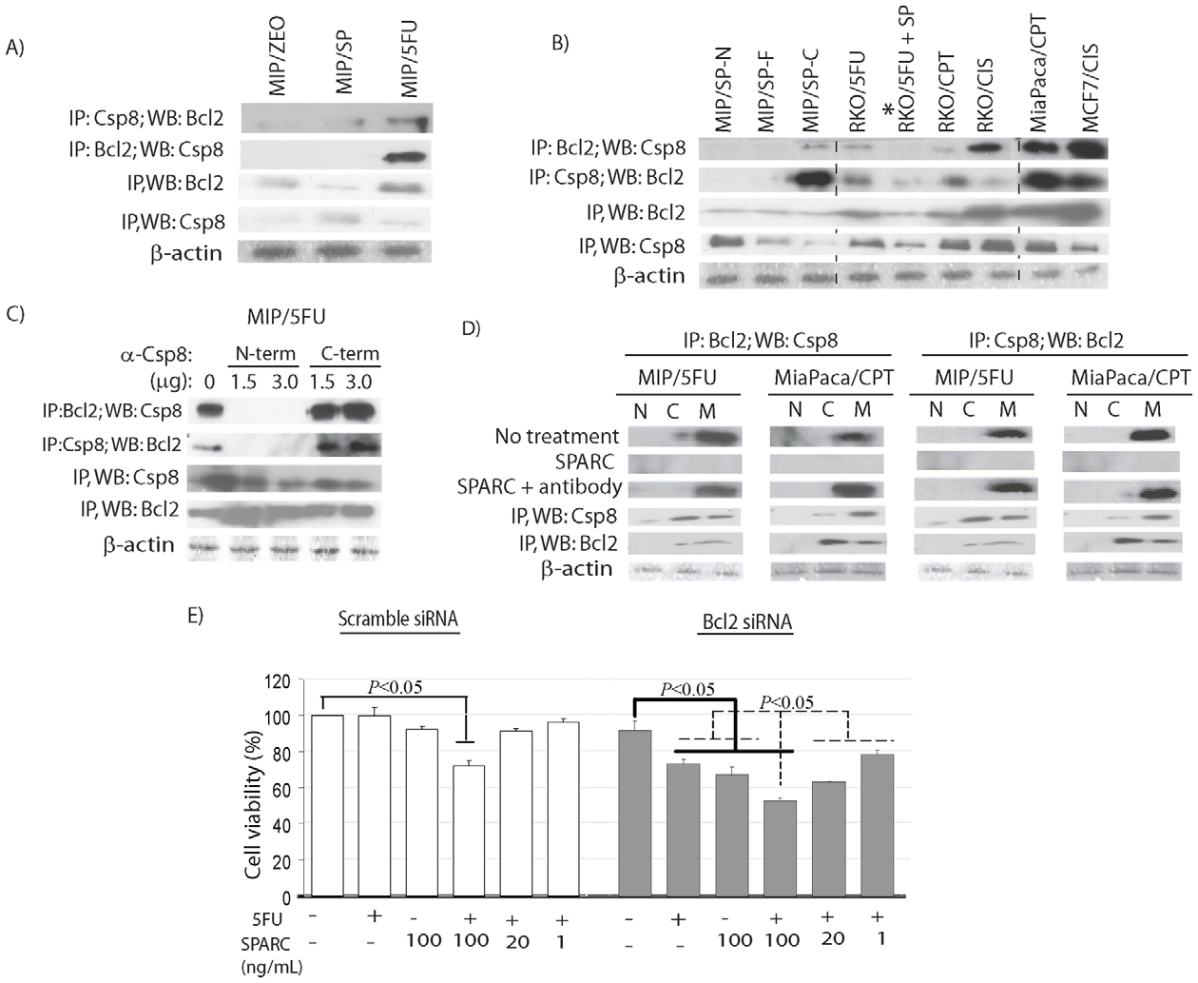
SPARC and Bcl2 both interact with caspase 8 with opposing effects. (**A**) In resistant MIP/5FU cells, caspase 8 interacts with Bcl2 and this is also observed in other resistant colorectal (RKO/5FU, RKO/CPT, RKO/CIS), pancreatic (MiaPaca/CPT) and breast cancer (MCF/CIS) cell lines (**B**). The Bcl2-caspase 8 interaction can be eliminated following exposure to rSPARC (100 ng/mL) (denoted as “*”). **C**) The interaction between Bcl2-caspase 8 occurs at the N-terminus of caspase 8 as cells incubated with antibodies blocking the N-terminus of caspase 8 (N-term, 1.5–3 µg) prevented this Bcl2-caspase 8 interaction. **D**) Bcl2-caspase 8 interaction is abolished after exposure to 100 ng/mL rSPARC in resistant MIP/5FU and MiaPaca/CPT cells. (**E**) Chemoresistant MIP/5FU cells transfected with Bcl2 siRNA to reduce Bcl2 expression are now responsive to 1000 µM 5-FU (+), especially in combination with incremental concentrations of rSPARC (20–100 ng/ml). Results represent mean ± s.e. (n = 3 independent studies). Student's t-test, statistically significant when p<0.05.

Our finding of an interaction between caspase 8 and SPARC, and previous reports of an interaction between caspase 8 and Bcl2 in neuroblastomas [17], led us to examine if such an interaction also occurs in cancer cells and whether it can be influenced by SPARC to facilitate apoptosis in response to chemotherapy. Our findings were interesting in that an interaction between caspase 8 and Bcl2 was detected in chemoresistant MIP/5FU cells, but not in sensitive MIP/ZEO and MIP/SP cells (Fig. 5A). This pattern of interaction was replicated in other chemotherapy-resistant cancer cells (Fig. 5B): CRC RKO/5FU, RKO/CPT, RKO/CIS; pancreatic MiaPaca/CPT; and breast cancer MCF-7/CIS. Even more exciting is the observation that when RKO/5FU cells were incubated with recombinant SPARC (rSPARC), the interaction between caspase 8 and Bcl2 was abolished (denoted by “*”, Fig. 5B).

Based on these observations, we proceeded to determine the region of caspase 8 that interacts with Bcl2, by incubating chemotherapy-resistant cells with antibodies targeting either the N- or C-terminus of caspase 8, and observed that the interaction could only be abolished when antibodies blocked the N-terminus of caspase 8 (Fig. 5C, Figure S2C). In addition, this caspase 8-Bcl2 interaction appeared to localize to the membrane fraction, and in line with previous results, was eliminated following exposure to rSPARC (Fig. 5D). However, co-incubation with rSPARC in the presence of anti-SPARC antibodies again restored this caspase 8-Bcl2 interaction (Fig. 5D).

These observations indicate that interactions involving Bcl2, caspase 8 and SPARC exist in cancer cells. In order to assess the significance of the interaction, the expression of Bcl2 was modulated by decreasing its expression in chemoresistant MIP/5FU cells. Only following a reduction in Bcl2 expression by siRNA transfection was there a decrease in cell viability in response to 5-FU (p = 0.04) or SPARC alone (p = 0.003), and these results were even more significant when the treatment included a combination of 5-FU with escalating concentrations of SPARC (Fig. 5E). These results support the biological importance of a Bcl2-caspase 8-SPARC interaction in modulating chemosensitivity.

### A synthetic peptide of the N-terminus of SPARC enhances apoptosis by inhibiting the interaction between caspase 8 and Bcl2

**Figure 6:**
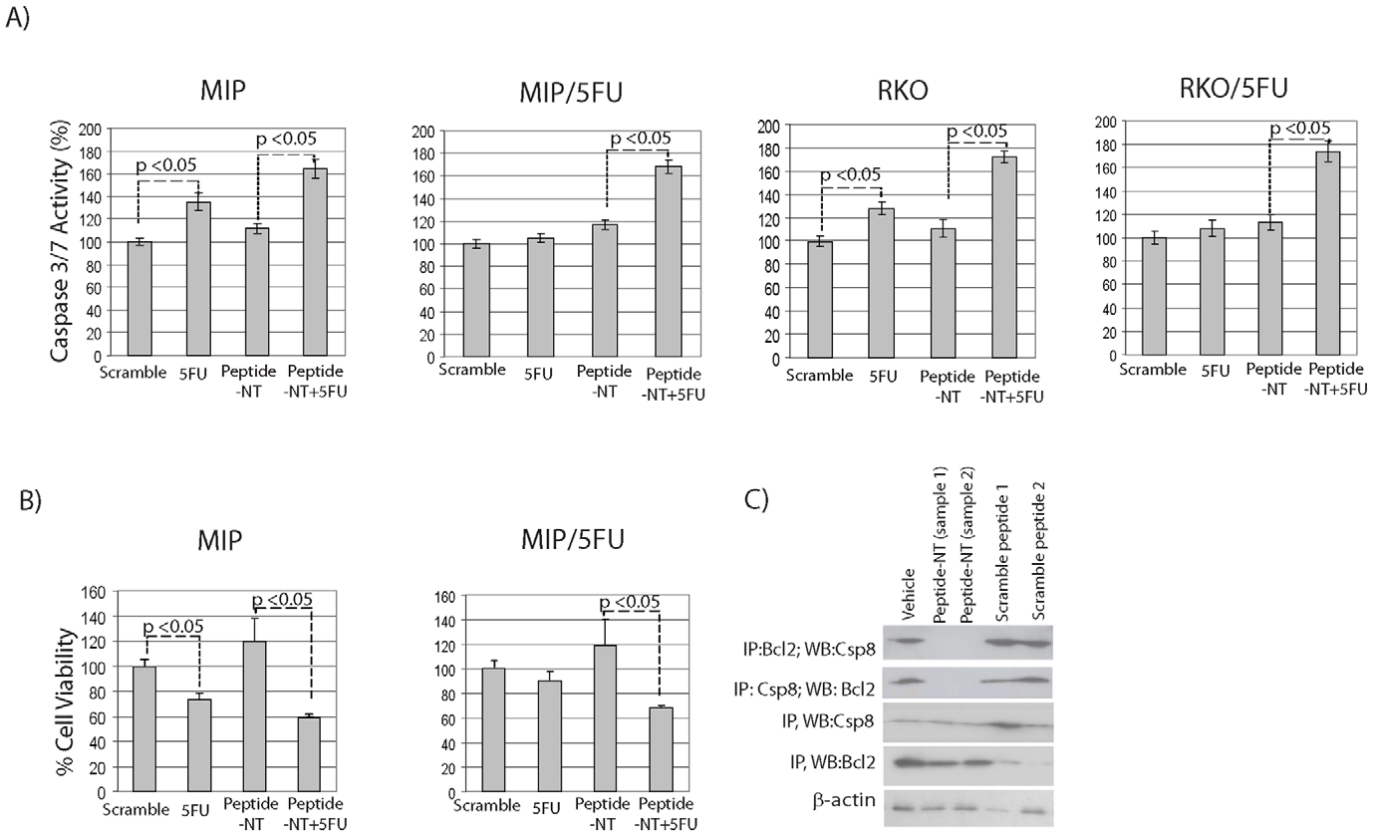
Augmentation of apoptosis by peptide-NT. Sensitive MIP101 or RKO and 5-FU resistant MIP/5FU and RKO/5FU cells were incubated with peptide-NT 100 ng/ml +/− 5-FU 5 µM for 24 hrs and assayed for (**A**) caspase 3/7 levels, or (**B**) cell viability by MTT assays. (**C**) Immunoblots showing the ability of peptide-NT to interfere with the interaction between caspase 8 and Bcl2: MIP/5FU cells were incubated with peptide-NT or scramble control peptides 1 or 2 for 24 hrs; cell pellets were collected 24 hrs later for co-immunoprecipitation studies.

In order to confirm that the N-terminus of SPARC did indeed confer greater chemosensitivity by augmenting apoptosis by interfering with the interaction between caspase 8 and Bcl2, we generated a synthetic peptide of the N-terminus of SPARC (peptide-NT), tagged with a TAT peptide (YGRKKRRQRRR) to facilitate its intracellular uptake. Caspase 3/7 assays confirmed ∼40–60% increase in apoptotic activity in not only sensitive MIP and RKO CRC cells, but as well the resistant MIP/5FU and RKO/5FU cells following incubation with peptide-NT and 5 µM 5-FU for 24 hrs (Fig. 6A). Also in line with previous observations: (1) cell viability also diminished significantly following exposure to this novel combination (Fig. 6B), and (2) immunoprecipitation studies revealed peptide-NT's ability to disrupt the interaction between caspase 8 and Bcl2 (Fig. 6C). These exciting results not only support our findings that the apoptosis-conferring region of SPARC resides within its N-terminus, but as well, that a synthetic peptide of this region is capable of chemosensitizing therapy-refractory cells.

### Mutations in the DED-domains of caspase 8 prevent interactions with Bcl2 and SPARC

**Figure 7:**
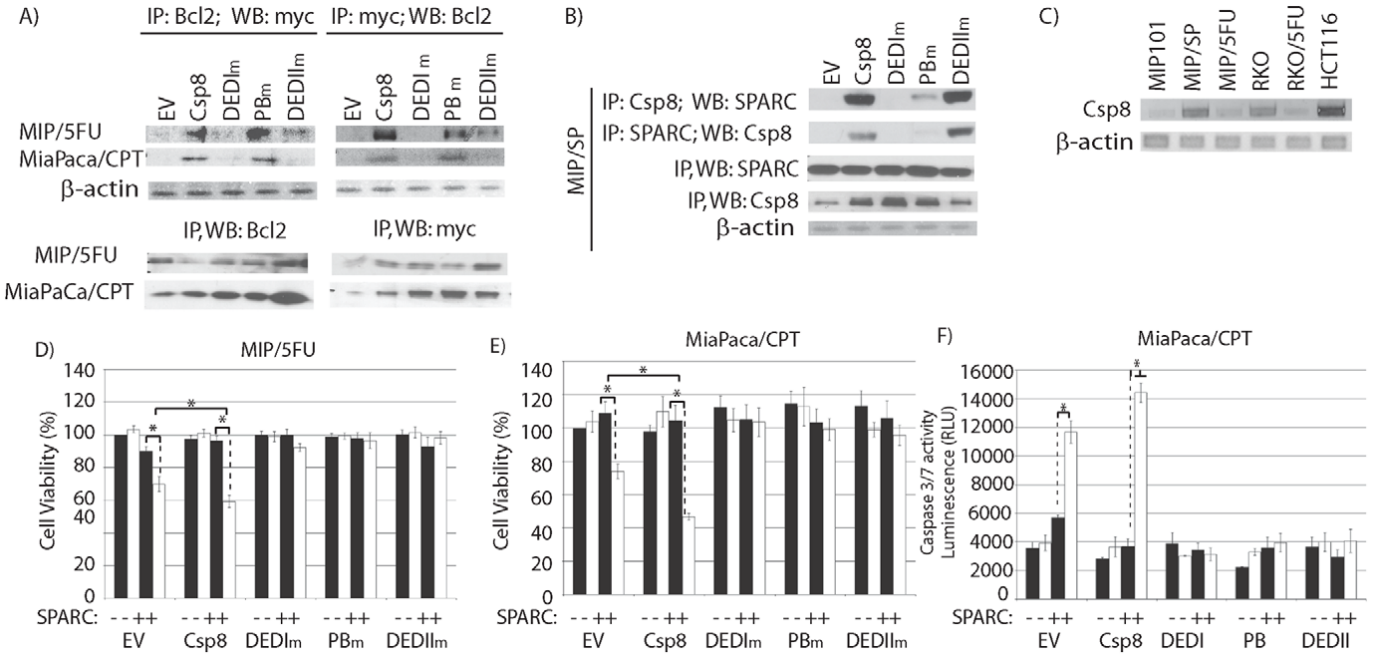
The DEDI-domain of caspase 8 is critical for its interaction with Bcl2. Vectors containing mutations in specific domains (DEDI, putative binding(PB), DEDII) of caspase 8 were transiently transfected into (**A**) MIP/5FU, MiaPaca/CPT or (**B**) MIP/SP cells, to determine the ability of the mutant proteins to co-IP with Bcl2 or SPARC. **C**) Basal levels of *caspase 8* gene expression between various cancer cell lines. **D–F**) The effect of SPARC on cell viability (**D, E**) or apoptosis (**F**) in cells overexpressing mutant forms of caspase 8 was assessed. Cells were transiently transfected with wild-type caspase 8 (Csp8), mutants (DEDIm, PBm, and DEDIIm), or empty-vector (EV, control) for 48 h, followed by exposure to 100 ng/mL rSPARC for 48 h, and treated with 0 (▪) or 500 µM (□)5-FU for an additional 24 h. **D–E**) In resistant cells, exposure to rSPARC and 5-FU resulted in decreased cell viability following transfection with EV-control: MIP/5FU: 90.14±2.80 (SPARC) vs. 69.89±4.64% (SPARC+5-FU) (p<0.005); MiaPaca/CPT: 108.77±6.85 (SPARC) vs. 73.98±4.46% (SPARC+5-FU) (p<0.005). Over-expression of wild-type caspase 8 in resistant cells further decreased cell viability following exposure to rSPARC and 5-FU, in comparison to EV-controls: MIP/5FU: 69.89±4.64 (SPARC + 5-FU) vs. 59.21±3.66% (SPARC + 5-FU + caspase 8) (p = 0.03); MiaPaca/CPT: 73.98±4.46 (SPARC + 5-FU) vs. 46.78±2.01% (SPARC + 5-FU + caspase 8) (p<0.005). However, over-expression of mutant forms of caspase 8 abolished the reduction in cell viability seen in the presence of rSPARC. Results represent mean ± s.e. (n = 3 independent studies). *p<0.05, Student's t-test.

**Figure 8:**
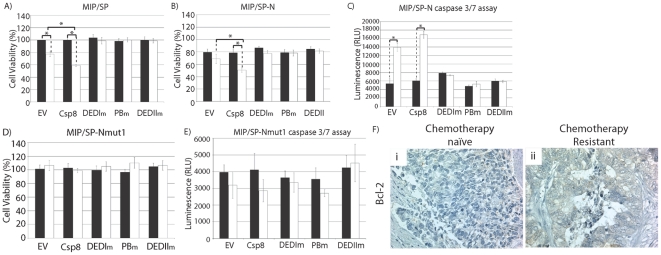
Interaction between the N-terminus of both caspase 8 and SPARC are required to reduce cell viability and enhance apoptosis. **A–B**) Cell viability further decreased in MIP/SP and MIP/SP-N after transfection with wild-type caspase 8 and 5-FU treatment, relative to empty vector (EV, control): MIP/SP: 77.2±3.8 (EV) vs. 59.1±1.5% (caspase 8) (p<0.005); MIP/SP-N: 68.8±4.8 (EV) vs. 50.6±3.6% (caspase 8) (p = 0.04). Transfection with any of the caspase 8 mutants eliminated their response to 5-FU, not only in terms of cell viability, but apoptosis (**C**). In cells overexpressing mutant SP-N (MIP/SP-Nmut1), transfection with caspase 8 was incapable of decreasing cell viability (**D**) or increasing apoptosis (**E**). Results represent mean ± s.e. (n = 3 independent studies). *p<0.05, Student's t-test. **F**) Bcl2 expression of clinical specimens of human CRCs obtained from the same individual following primary tumor resection (chemotherapy-naïve) and disease recurrence post-chemotherapy (chemotherapy-resistant) (paraffin-embedded 6 µM sections).

To identify the specific site within caspase 8 that interacts with Bcl2 or SPARC, mutations were introduced in three different regions of the N-terminus of caspase 8: the DEDI-domain (DEDIm), the putative binding region (PBm), and DEDII-domain (DEDIIm). Co-immunoprecipitation studies revealed that transient overexpression of DEDIm and DEDIIm caspase 8 mutants interfered with the ability of Bcl2 to interact with caspase 8 in MIP/5FU and MiaPaca/CPT cells (Fig. 7A). In MIP/SP cells, overexpression of DEDIm-caspase 8 eliminated the interaction between SPARC-caspase 8, while mutations in the PB or DEDII-domains still allowed caspase 8 to interact with SPARC (Fig. 7B). These results suggest that the DEDI-domain of caspase 8 is the site of interaction with SPARC and Bcl2. Interestingly, we also noted that over-expression of caspase 8-mutants abolished the reduction in cell viability and increase in apopotosis that is normally conferred by caspase 8 in the presence of SPARC and 5-FU, in resistant cells (Fig. 7D–F). Similar observations were noted with chemosensitive MIP/SP and MIP/SP-N cells (Fig. 8A–C). Also, MIP/SP-Nmut cells failed to respond to 5-FU following transfection with either wild-type or mutant caspase 8 (Fig. 8D,E), thus supporting the N-terminus of SPARC as the site of interaction with caspase 8.

The results reported in this study reveal how an interplay between SPARC, caspase 8 and Bcl2 may modulate apoptosis in response to cytotoxic agents. In addition, higher Bcl2 levels in human CRC (Fig. 8F) suggest that the ability of SPARC to interfere with Bcl2's interaction with caspase 8 may be clinically relevant and can be targeted as a potential therapeutic.

## Discussion

Apoptosis occurs as a result of a series of well coordinated events that classically involve either activation of the extrinsic or intrinsic pathways and members of the Bcl2 protein family. We and others have previously shown that high levels of SPARC, a matricellular protein known to influence cell growth and apoptosis, to be associated with increased apoptosis in ovarian, pancreatic and CRCs [4], [6], [7], and that its exogenous exposure in-vivo promotes greater tumor regression in CRCs that had become refractory to conventional chemotherapies [4]. Recently, we identified a potential mechanism that allows SPARC to augment the apoptotic cascade, enhancing the effect of cytotoxic agents used in the treatment of malignancies [8]: SPARC promotes the activation of the extrinsic pathway of apoptosis by interacting with the N-terminus of caspase 8, with subsequent involvement of the intrinsic pathway, via Bid, to enhance apoptosis [8]. As an extension to these earlier observations, the current study demonstrates that in cancer cells that have become resistant to chemotherapy, the N-terminus of caspase 8 interacts instead with Bcl2 to restrict apoptosis. However, this inhibitory effect on apoptosis is reversible in the presence of higher levels of SPARC, either following exogenous exposure to this protein or its forced endogenous over-expression. Importantly, we not only identify the site interacting with caspase 8 as the N-terminus domain of SPARC, but that a synthetic peptide of this region (51aa in length) is responsible for conferring SPARC's apoptotic activity. In fact, our results demonstrate that tumor xenografts of cells over-expressing only the N-terminus domain of SPARC, and not others, experienced the most dramatic tumor regression in response to chemotherapy.

SPARC is expressed in many different cell types [18] and plays a complex role in tumorigenesis. While some studies report a positive association between high SPARC and more aggressive tumors [19], [20], several studies, including our own, support the view that it functions in part as a tumor suppressor in neuroblastomas, leukemias, colorectal, ovarian, pancreatic, lung, and breast cancers [3], [4], [6], [7], [21], [22]. These divergent actions of SPARC are puzzling, but may be explained by differences in the biological activities of the various proteolytic products of SPARC. Specifically, previous studies have demonstrated that SPARC undergoes proteolysis by a variety of proteases, such as metalloproteinases (MMPs), elastases, cathepsins, and serine proteases [23]. These degradation products have differing biological activities [10]: for example, a small peptide containing only 20 amino acids (aa 21–40) of the N-terminus domain of SPARC had previously been shown to inhibit endothelial cell spreading and bFGF-induced cell migration [24]; and the native SPARC protein is also known for its ability to inhibit angiogenesis [10], yet release of a proteolytic product containing the Cu^2+^ binding sequence KGHK (lysine-glycine-histidine-lysine) opposes the activity of the full-length protein by instead, stimulating angiogenesis in-vitro and in-vivo [25], [26]. Therefore, differences in tumor-specific protease expression may account for the differences in SPARC's biological behavior in tumorigenesis and different cancers. This also helps explain our findings that the effect of the recombinant peptide of SPARC's N-terminus domain on tumor regression was superior to SPARC: in addition to enhancing apoptosis in response to 5-FU, it also reduced the rate of tumor growth by ∼ 40% in comparison to SPARC-over-expressing tumors. Our findings indicate that while SPARC's N-terminus domain inhibits tumor growth, the follistatin-like (FS-) domain alone may have growth-promoting properties, as xenografts overexpressing recombinant proteins of this fragment had significantly shorter doubling times than control xenografts. These tumors were also unresponsive to 5-FU treatment. Taken together, these observations suggest that the efficacy of the native SPARC protein on tumor regression may be blunted when its proteolysis results in the release of different fragments with opposing biological effects. These observations again reflect SPARC's intricate biology, as potential proteolytic products may have opposing effects on tumor growth, progression and response to therapy. This is an area that requires further investigations, in order to allow a better understanding of SPARC's effect in different types of cancers.

**Figure 9:**
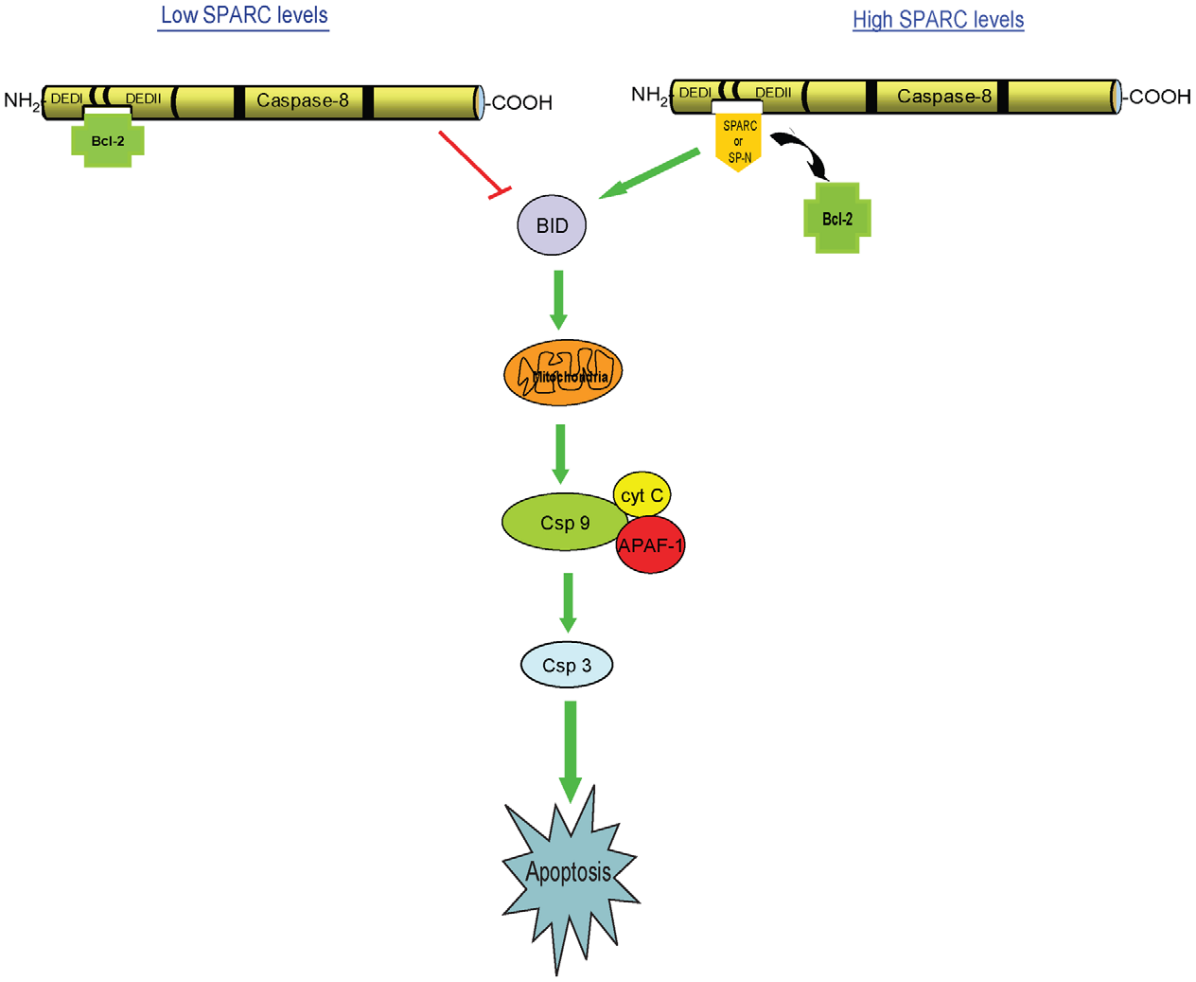
A model: SPARC-mediated apoptosis. A schematic of SPARC and Bcl2 interacting with caspase 8 to influence apoptosis. In this study, we demonstrate that in an environment low in SPARC, Bcl2 interacts with caspase 8 to inhibit apoptosis. However, in the presence of SPARC or its N-terminus, these proteins interact with caspase 8 to inhibit its interaction with Bcl2, leading to an augmentation of the apoptotic cascade.

Based on the results of the current study, another dimension has been added to the multifaceted biological behavior of SPARC, by demonstrating the pro-apoptotic activity of the N-terminus domain of SPARC. In addition, we further confirm the involvement of Bid in SPARC-mediated apoptosis as cells overexpressing only the N-terminus of SPARC (MIP/SP-N) are capable of activating Bid, and this effect was easily abolished with Bid-siRNA in MIP/SP-N cells only. More importantly, we show that the ability of either SPARC or its smaller N-terminus domain to promote apoptosis results from their interference in Bcl2's interaction with caspase 8, thereby leading to the activation of the extrinsic pathway (Fig. 9). Our assessment of a potential triad involving SPARC-caspase 8-Bcl2 in regulating apoptosis was based on earlier observations that Bcl2 binds to caspase 8 to inhibit caspase 8-mediated apoptosis in Fas-resistant neuroblastomas [17]; reports of SPARC's ability to promote apoptosis by decreasing the ratio of Bcl2 and BAX in autosomal dominant polycystic kidney cells [27], together with our recent observations that decreasing SPARC expression by siRNA diminishes chemosensitivity [28] and that this effect is based on SPARC's ability to interact with caspase 8 to promote apoptosis [8]. Bcl2 has long been associated with drug resistance since its discovery as a proto-oncogene in non-Hodgkin's B-cell lymphomas [29], and over the years, it has been extensively studied as a potential target for cancer therapy [30]. Recently, the differential compartmentalization of Bcl2 and NRAS has been reported to influence disease states, such as myelodysplastic syndromes and acute myeloid leukemia [31]. In their mouse model, the physical interaction of Bcl2 and mutant NRAS affected the apoptotic machinery. These novel interactions between Bcl2 and other proteins, as demonstrated by us and others, highlight the complex mechanisms utilized by cancer cells to evade cell death, leading to disease progression and drug resistance.

In this study, we demonstrate that Bcl2 and caspase 8 interact in cancer cells that have become refractory to therapy, thus providing another mechanism by which Bcl2 promotes survival and drug resistance. In addition, our in-vitro and in-vivo results reveal that the N-terminus domain of SPARC, and its synthetic peptide, are highly efficacious in modulating and enhancing apoptosis, in part, through its ability to dissociate the interaction between Bcl2 and caspase 8. These exciting findings point to the possibility that this smaller N-terminus peptide, based on its ability to interfere with the Bcl2-caspase 8 interaction, can be exploited as a potential therapeutic in disease states, such as cancer, where augmentation of the apoptotic cascade is clinically beneficial.

## Materials and Methods

### Cell Lines, Reagents

Colorectal (MIP101, HCT 116, RKO), pancreatic (MiaPaCa) and breast (MCF-7) cancer cells (sensitive and resistant) were maintained in DMEM media as previously described [4]. HCT116, RKO, MiaPaCa and MCF-7 were obtained from ATCC. MIP101 cells were kind gifts from Dr. Lan Bo Chen [32]. Also, please refer to Table S1 for a list of abbreviations. For resistant cells, media were also supplemented as follows: MIP/5FU, 500 µM 5-FU; MIP/CPT, 10 µM CPT-11; HCT116/5FU, 10 µM 5-FU; RKO/5FU, 25 µM 5-FU; RKO/CPT, 18 µM CPT-11; cisplatin (CIS) (RKO/CIS), 30 µM CIS; MiaPaca/CPT, 100 µM CPT-11; MCF7/CIS, 30 µM CIS. MIP101 transfected with empty vector (MIP/ZEO) or stably transfected with SPARC (MIP/SP) underwent selection with Zeocin (Invitrogen).

Mammalian expression vectors containing the N-terminus (SP-N), follistatin-like (SP-F), and extracellular (SP-C) domains of SPARC were kind gifts from Dr. W. Schiemann (National Jewish Medical and Research Centre; Denver, CO). The recombinant proteins were myc/His_6_–tagged [33]. MIP101 cells stably transfected with SP-N (MIP/SP-N), SP-F (MIP/SP-F), or SP-C (MIP/SP-C) were also generated and selected for hygromycin-resistance. Overexpression of these recombinant proteins in cells were confirmed by ELISA (Figure S3). Of note, previous studies utilizing tagged-SPARC (or related peptides) have demonstrated similar biological activity as the native, un-tagged proteins [33], [34], therefore, myc or His_6_–tagged proteins were used in these studies.

Recombinant human SPARC (rSPARC), were generously provided by Dr. Desai (Abraxis BioScience Inc., USA). Human peptide of the N-terminus domain of SPARC (Peptide sequence: APQQEALPDETEVVEETVAEVTEVSVGANPVQVEVGEFDDGAEETEEEVVA) was synthesized by AnaSpec (USA) with a TAT sequence (YGRKKRRQRRR) added to its amino-terminus, to facilitate its intracellular entry.

### Site-directed Mutagenesis

SPARC (SP), SP-N, SP-F, and caspase 8 containing plasmids were used for site-directed mutagenesis, using GeneTailor Site-Directed Mutagenesis System (Invitrogen) as per manufacturer's protocol. Mutant constructs were verified by DNA sequencing. Please refer to Table S2 for a list of primers used.

### Transfection studies

#### Plasmids

In order to assess if the smaller peptides representing the different domains of SPARC had an effect on cell viability, cells were transiently transfected with constructs containing: SPARC (SP), SPARC's N-terminus domain (SP-N), mutant FS-domain (SP-F), EC domain (SP-C); mutant N-terminus domain (SP-Nmut1, SP-Nmut2), FS-domain (SP-Fmut1, SP-Fmut2); wild-type caspase 8 or mutant DEDI-domain (DEDIm), putative-binding-domain (PBm), DEDII-domain (DEDIIm); or empty-vector control (ZEO) for 48-hours. Depending on the study, cells were then exposed to 100 ng/mL SPARC, and/or 5–1000 µM 5-FU (MIP101-related cells; with the higher concentrations of 5-FU used in the resistant MIP/5FU cells as their IC_50_ was >18-fold higher than sensitive MIP101 cells), 50–100 µM CPT-11 (MIP101 related cells), 18–36 µM CPT-11 (RKO-related cells), 100–200 µM CPT-11 (MiaPaca-related cells), or 60 µM CIS (MCF7-related cells) for 24–48-hours. Stable cell lines overexpressing the different SPARC domains were also generated and subjected to similar treatment protocols. Cell viability and proliferation were assessed by WST reagent (Roche) at 450 nM [8] at the completion of the study.

#### RNA Interference

Caspase 8 gene expression knockdown using 40 nM caspase 8 siRNA (Stealth RNAi, Invitrogen) was assessed as previously described, using similar conditions that resulted in a 14-fold reduction in gene expression [8]. Transfection with siRNA was performed for 48-hrs before additional treatments or assays were performed. The conditions for Bid siRNA transfection were tested using 20–40 nM siRNA (Stealth RNAi. Invitrogen) and the degree of knock-down determined 48 and 72 hrs later (Figure S2A). The most effective knock-down was obtained following transfection with 40 nM of Bid siRNA for either 48 or 72 hrs. All subsequent experiments were performed using 40 nM siRNA for 48 hrs. For Bcl2, conditions were optimized using 40–80 nM siRNA (Stealth RNAi, Invitrogen) and 4.5 uL of HiperFect-Transfection Reagent (Qiagen) for 48–72 hrs. At 72 hr following transfection with 40 and 80 nM of siRNA, a 40% and 60% reduction in Bcl2 expression was achieved, respectively (Figure S2B). In subsequent experiments, 80 nM Bcl2 siRNA for 72 hrs were used. As control, scramble oligonucleotide sequences were used. For RT-PCR, RNA was isolated with Trizol (Invitrogen), and 1 µg of total RNA was used to generate cDNA (Superscript III, Invitrogen). Specific primers used for PCR include: Bcl2 (forward)5′-atctgggccacaagtgaagt-3′, (reverse)5′-cttctccccagcctccag-3′; Bid (forward)5′-aaaaccacatggcacagaga-3′, (reverse)5′-agagggaaccactttgctga-3′; and under the following PCR conditions: 94°C×2 min; 35 cycles of 94°C×30 s, 58°C×20 s, 72°C×30 s; 72°C×30 s, PCR products were separated on a 1.5% agarose gel electrophoresis, and levels of expression quantified using ImageJ.

### ELISA for quantification of SPARC and related peptides

Stable cell lines (MIP/ZEO, MIP/SP, MIP/SP-N, MIP/SP-F and MIP/SP-C) or transiently transfected cells were grown to 90% confluence and proteins from whole cell lysates were isolated as previously described [4]. Microwells were coated with anti-SPARC antibody (1∶5000) in coating buffer (0.1 M sodium carbonate, pH 9.5) overnight at 4°C, washed 3× with PBS-T (0.05% Tween-20), and blocked with 2%BSA in PBS for 1-hour at RT. 5 µg of total protein was added to each well in triplicate, incubated at 4°C for 4-hrs, and washed 3× with PBS-T. Wells were then incubated with either anti-His or anti-myc (1∶5000, Sigma; all expression constructs express His_6_/myc-tagged fusion proteins) antibodies for 2-hours at RT, washed 5× with PBS-T, and incubated with appropriate secondary-HRP antibodies (1∶10 000) for 1-hour at RT. Wells were then washed 5× with PBS-T and 100 µL of TMD chromagen substrate was added to each well for 15–30-minutes. Absorbance was read at 650 nM. Results of the ELISA are provided in Figure S3.

### Apoptosis

#### M30 Antibody staining

Cells were seeded and treated 48 hours later with 5 µM 5-FU for an additional 48 hours, harvested and fixed onto glass slides by Cytospin, and stained with M30 antibodies (M30 CytoDEATH monoclonal antibody, ALEXIS Biochemicals, Switzerland) as per manufacturer's protocol.

#### Caspase- 3/7 assay

20 µg of total protein isolated from cell lysates were used in Caspase-Glo 3/7 Assay (Promega) as previously described [35]. The relative luminescence units (RLU) were measured using Viktor^2^ 1320 Multilabel-platform (Perkin Elmer).

### Immunoblot analysis

48 hours after plating, cells were incubated with 5 µM 5-FU, and collected at 0–12 hours for immunoblotting as previously described [4], [36]. Immunodetection was performed using antibodies against caspase 8 (1∶1000, Santa Cruz Biotechnology, Inc) or cleaved-caspase 8, BID, and caspase 3 (all 1∶1000, Cell Signaling Technologies) followed by incubation with the appropriate secondary antibody. All immunoblots were also probed with antibodies to β-actin (0.32 µg/mL, Abcam) as loading control. Proteins were detected with SuperSignal West Dura (Pierce).

### Subcellular Fractionation, Co-Immunoprecipitation

Total protein was isolated from cells grown to ∼80% confluence, then separated into nuclear, cytosolic, and membrane fractions as previously described [8]. 250 µg of individual cellular fractions were incubated with antibodies against His_6_ or myc (1∶100), caspase 8 (N-terminus, 1∶100, Abcam), Bcl2 (1∶100, Cell Signaling Technologies) or a non-specific anti-mouse-IgG antibody as control (Cell Signaling Technologies), in PBS overnight (4°C). Protein∶Antibody mixture was then incubated with 30 µL of Protein A∶Protein G beads (1∶1,Sigma) for 4-hours (4°C), washed with PBS, and eluted with 2× SDS-loading buffer, and used for immunoblotting against caspase 8, His_6_, or Bcl2 [8].

### Animal Studies

Tumor xenograft animal models were used to assess how overexpression of the various fragments of SPARC could influence tumor progression and response to chemotherapy in-vivo. Nude mice were implanted with 1×10^6^ (MIP/ZEO, MIP/SP, MIP/SP-N, MIP/SP-F, or MIP/SP-C) cells into the flanks of each animal. Treatment with 5-FU using a 3-week cycle regimen (6 cycles), commenced once the average tumor size reached 75–100 mm^3^ [4]. Control animals received saline. All studies were approved by the Animal Care Committee at the University of British Columbia, Canada (protocol A06-1507). Doubling time was averaged from all tumor measurements and calculated as: t_1/2_ = (t_2_-t_1_)*ln(2)/ln(volume_2_/volume_1_). In order to minimize differences in tumor engraftment as a potential reason for influencing the variability in cell doubling time, all xenografts were implanted with the same number of cells and only mice with tumors ∼75–100 mm^3^ in size by 2 weeks following implantation were used in the in-vivo studies.

#### Immunohistochemistry

For caspase 8 (1∶100), Bcl2 (1∶100), and SPARC (1∶100, Hematological technologies) expression, OCT-embedded tissues were sectioned and processed according to established protocols [4], [36]; counterstained with TO-PRO-3 iodide (Invitrogen). Confocal Zeiss-Nikon microscope was used for image capture and Olympus BX61 microscope for light microscopy.

#### CD31 staining

Tumors embedded in OCT-media were sectioned and fixed in acetone for 10-minutes at 4°C, washed in 1× PBS, blocked in 2% NCS for 20-minutes at RT, and incubated in CD31 antibody (1∶100, DAKO) overnight at 4°C. Sections were counterstained with DAPI (Molecular probes). Zeiss-Axioplan2 fluorescence microscope was used for imaging. The number of CD31-positive cells were counted and averaged from three different fields (n = 3 independent tumors).

Routine histology was also performed on paraffin-embedded tissues (6 µM thick) [8] for hematoxyline and eosin (H&E) or cleaved-caspase 3 staining (1∶25 dilution). Leica MicroSystems Bond-Max was used to stain tissue slides with cleaved-caspase 3 based on the manufacturer's protocols with the following modifications: Leica's ER1 Solution (citrate based buffer) was used for antigen retrieval (10-min×100°C), followed by a 10-min peroxide block at RT. Cleaved-caspase 3 antibody was incubated overnight at 4°C, then stained with hematoxylin. H&E images were captured with a Zeiss Axioplan 2 microscope, while images of caspase 3 stained slides were analyzed with a Leica DM600B microsope and Surveyor (version 5.5.5.12).

## Supporting Information

Figure S1 **Histology of tumor xenografts.** Tumor xenografts of MIP/ZEO, MIP/SP, MIP/SP-N, MIP/SP-F, and MIP/SP-C subcutaneously implanted cells were paraffin-embedded and processed and stained with Hematoxylin and Eosin (H&E), and cleaved caspase 3.
(TIF)

Figure S2 **siRNA optimization for Bid and Bcl2; and interaction between Bcl2 and caspase 8 in various resistant cells.** **A**) Bid siRNA optimization: MIP101 cells were transfected with 20–40 nM Bid siRNA (or scramble control). RNA was isolated from cells harvested after 48–72 hrs of transfection and Bid gene expression determined by RT-PCR. Optimal Bid gene expression knock-down was achieved after transfection of 40 nM of siRNA for either 48–72 hrs; **B**) Bcl2 siRNA optimization: MIP/5FU cells were transfected with 40–80 nM Bcl2 siRNA (or scramble control) for 72 hrs. A 40% and 60% reduction in Bcl2 gene expression was noted following transfection with 40 nM and 80 nM respectively. The optimal condition used in subsequent experiments included the transfection of cells with 80 nM of Bcl2 siRNA for 72 hrs; **C**) The interaction between Bcl2-caspase 8 occurs at the N-terminus of caspase 8 as cells incubated with antibodies blocking caspase 8 (N-term, 1.5–3 µg) prevented this Bcl2-caspase 8 interaction.
(TIF)

Figure S3 **Levels of SPARC and related peptides in cells used in this study.** Cell lysates from stable transfectants (in-vitro and in-vivo) or transiently transfected cells were isolated 120 hours post-transfection and levels of SPARC and SPARC-related peptide levels were assayed by ELISA. Results represent mean ± s.e. (n = 3 independent studies). Student's t-test, * statistical difference compared to control, where p<0.05.
(TIF)

Table S1 **List of abbreviations.**
(DOC)

Table S2 **Site-directed mutagenesis primers.**
(DOC)

## References

[1] Chlenski A, Liu S, Crawford SE, Volpert OV, DeVries GH (2002). SPARC is a key Schwannian-derived inhibitor controlling neuroblastoma tumor angiogenesis.. Cancer Res.

[2] DiMartino JF, Lacayo NJ, Varadi M, Li L, Saraiya C (2006). Low or absent SPARC expression in acute myeloid leukemia with MLL rearrangements is associated with sensitivity to growth inhibition by exogenous SPARC protein.. Leukemia.

[3] Sato N, Fukushima N, Maehara N, Matsubayashi H, Koopmann J (2003). SPARC/osteonectin is a frequent target for aberrant methylation in pancreatic adenocarcinoma and a mediator of tumor-stromal interactions.. Oncogene.

[4] Tai IT, Dai M, Owen DA, Chen LB (2005). Genome-wide expression analysis of therapy-resistant tumors reveals SPARC as a novel target for cancer therapy.. J Clin Invest.

[5] Said N, Motamed K (2005). Absence of host-secreted protein acidic and rich in cysteine (SPARC) augments peritoneal ovarian carcinomatosis.. Am J Pathol.

[6] Puolakkainen PA, Brekken RA, Muneer S, Sage EH (2004). Enhanced growth of pancreatic tumors in SPARC-null mice is associated with decreased deposition of extracellular matrix and reduced tumor cell apoptosis.. Mol Cancer Res.

[7] Yiu GK, Chan WY, Ng SW, Chan PS, Cheung KK (2001). SPARC (secreted protein acidic and rich in cysteine) induces apoptosis in ovarian cancer cells.. Am J Pathol.

[8] Tang MJ, Tai IT (2007). A novel interaction between procaspase 8 and SPARC enhances apoptosis and potentiates chemotherapy sensitivity in colorectal cancers.. J Biol Chem.

[9] Bradshaw AD, Reed MJ, Carbon JG, Pinney E, Brekken RA (2001). Increased fibrovascular invasion of subcutaneous polyvinyl alcohol sponges in SPARC-null mice.. Wound Repair Regen.

[10] Tai IT, Tang MJ (2008). SPARC in cancer biology: its role in cancer progression and potential for therapy.. Drug Resist Updat.

[11] Lane TF, Sage EH (1990). Functional mapping of SPARC: peptides from two distinct Ca+(+)-binding sites modulate cell shape.. J Cell Biol.

[12] Funk SE, Sage EH (1991). The Ca2(+)-binding glycoprotein SPARC modulates cell cycle progression in bovine aortic endothelial cells.. Proc Natl Acad Sci U S A.

[13] Chlenski A, Liu S, Baker LJ, Yang Q, Tian Y (2004). Neuroblastoma angiogenesis is inhibited with a folded synthetic molecule corresponding to the epidermal growth factor-like module of the follistatin domain of SPARC.. Cancer Res.

[14] Maurer P, Hohenadl C, Hohenester E, Gohring W, Timpl R (1995). The C-terminal portion of BM-40 (SPARC/osteonectin) is an autonomously folding and crystallisable domain that binds calcium and collagen IV.. J Mol Biol.

[15] Kupprion C, Motamed K, Sage EH (1998). SPARC (BM-40, osteonectin) inhibits the mitogenic effect of vascular endothelial growth factor on microvascular endothelial cells.. J Biol Chem.

[16] Chlenski A, Liu S, Guerrero LJ, Yang Q, Tian Y (2006). SPARC expression is associated with impaired tumor growth, inhibited angiogenesis and changes in the extracellular matrix.. Int J Cancer.

[17] Poulaki V, Mitsiades N, Romero ME, Tsokos M (2001). Fas-mediated apoptosis in neuroblastoma requires mitochondrial activation and is inhibited by FLICE inhibitor protein and Bcl-2.. Cancer Res.

[18] Wang W, Mei C, Tang B, Zhao H, Xu C (2006). Aberrant expression of SPARC and its impact on proliferation and apoptosis in ADPKD cyst-lining epithelia.. Nephrol Dial Transplant.

[19] Ledda MF, Adris S, Bravo AI, Kairiyama C, Bover L (1997). Suppression of SPARC expression by antisense RNA abrogates the tumorigenicity of human melanoma cells.. Nat Med.

[20] Schultz C, Lemke N, Ge S, Golembieski WA, Rempel SA (2002). Secreted protein acidic and rich in cysteine promotes glioma invasion and delays tumor growth in vivo.. Cancer Res.

[21] Gilles C, Bassuk JA, Pulyaeva H, Sage EH, Foidart JM (1998). SPARC/osteonectin induces matrix metalloproteinase 2 activation in human breast cancer cell lines.. Cancer Res.

[22] Rempel SA, Golembieski WA, Ge S, Lemke N, Elisevich K (1998). SPARC: a signal of astrocytic neoplastic transformation and reactive response in human primary and xenograft gliomas.. J Neuropathol Exp Neurol.

[23] Motamed K (1999). SPARC (osteonectin/BM-40).. Int J Biochem Cell Biol.

[24] Funk SE, Sage EH (1993). Differential effects of SPARC and cationic SPARC peptides on DNA synthesis by endothelial cells and fibroblasts.. J Cell Physiol.

[25] Lane TF, Iruela-Arispe ML, Johnson RS, Sage EH (1994). SPARC is a source of copper-binding peptides that stimulate angiogenesis.. J Cell Biol.

[26] Iruela-Arispe ML, Lane TF, Redmond D, Reilly M, Bolender RP (1995). Expression of SPARC during development of the chicken chorioallantoic membrane: evidence for regulated proteolysis in vivo.. Mol Biol Cell.

[27] Wang H, Workman G, Chen S, Barker TH, Ratner BD (2006). Secreted protein acidic and rich in cysteine (SPARC/osteonectin/BM-40) binds to fibrinogen fragments D and E, but not to native fibrinogen.. Matrix Biol.

[28] Cheetham S, Tang MJ, Mesak F, Kennecke H, Owen D (2008). SPARC promoter hypermethylation in colorectal cancers can be reversed by 5-Aza-2′deoxycytidine to increase SPARC expression and improve therapy response.. Br J Cancer.

[29] Tsujimoto Y, Cossman J, Jaffe E, Croce CM (1985). Involvement of the bcl-2 gene in human follicular lymphoma.. Science.

[30] Mohammad R, Giri A, Goustin AS (2008). Small-molecule inhibitors of Bcl-2 family proteins as therapeutic agents in cancer.. Recent Patents Anticancer Drug Discov.

[31] Omidvar N, Kogan S, Beurlet S, le Pogam C, Janin A (2007). BCL-2 and mutant NRAS interact physically and functionally in a mouse model of progressive myelodysplasia.. Cancer Res.

[32] Modica-Napolitano JS, Steele GD, Chen LB (1989). Aberrant mitochondria in two human colon carcinoma cell lines.. Cancer Res.

[33] Schiemann BJ, Neil JR, Schiemann WP (2003). SPARC inhibits epithelial cell proliferation in part through stimulation of the transforming growth factor-beta-signaling system.. Mol Biol Cell.

[34] Haber CL, Gottifredi V, Llera AS, Salvatierra E, Prada F (2008). SPARC modulates the proliferation of stromal but not melanoma cells unless endogenous SPARC expression is downregulated.. Int J Cancer.

[35] Chan JM, Ho SH, Tai IT (2010). Secreted protein acidic and rich in cysteine-induced cellular senescence in colorectal cancers in response to irinotecan is mediated by P53.. Carcinogenesis.

[36] Taghizadeh F, Tang MJ, Tai IT (2007). Synergism between vitamin D and secreted protein acidic and rich in cysteine-induced apoptosis and growth inhibition results in increased susceptibility of therapy-resistant colorectal cancer cells to chemotherapy.. Mol Cancer Ther.

