# Correction: A Peptide of SPARC Interferes with the Interaction between Caspase8 and Bcl2 to Resensitize Chemoresistant Tumors and Enhance Their Regression In Vivo

**DOI:** 10.1371/journal.pone.0127226

**Published:** 2015-04-23

**Authors:** Mahbuba Rahman, Annie P. K. Chan, Michelle J. Tang, Isabella T. Tai

The author list for this article should be corrected to list Dr. Michelle Tang as the third author. Michelle Tang’s inclusion as an author is in line with the recommendation of the University of British Columbia, which evaluated the contributions to this work.

The revised author list should thus read as follows:

Mahbuba Rahman, Annie P. K. Chan, Michelle J. Tang, Isabella T. Tai

The affiliation for Michelle J Tang at the time of the study was: Division of Gastroenterology, University of British Columbia, Vancouver, British Columbia, Canada; Genome Sciences Centre, British Columbia Cancer Agency, Vancouver, British Columbia, Canada

The correct citation is:

Rahman M, Chan APK, Tang MJ, Tai IT (2011) A Peptide of SPARC Interferes with the Interaction between Caspase8 and Bcl2 to Resensitize Chemoresistant Tumors and Enhance Their Regression In Vivo. PLoS ONE 6(11): e26390. doi:10.1371/journal.pone.0026390

The Authors Contributions should also be revised as below:

Conceived and designed the experiments: ITT, MT. Performed the experiments: MR APKC, MT. Analyzed the data: MR APKC ITT, MT. Contributed reagents/materials/analysis tools: ITT. Wrote the paper: ITT, MT.
