# Supplementary figures and images for: A Peptide of SPARC Interferes with the Interaction between Caspase8 and Bcl2 to Resensitize Chemoresistant Tumors and Enhance Their Regression In Vivo
Source: PLoS One. 2011 Nov 1;6(11):e26390. doi: 10.1371/journal.pone.0026390 (PMC3206029; doi:10.1371/journal.pone.0026390)

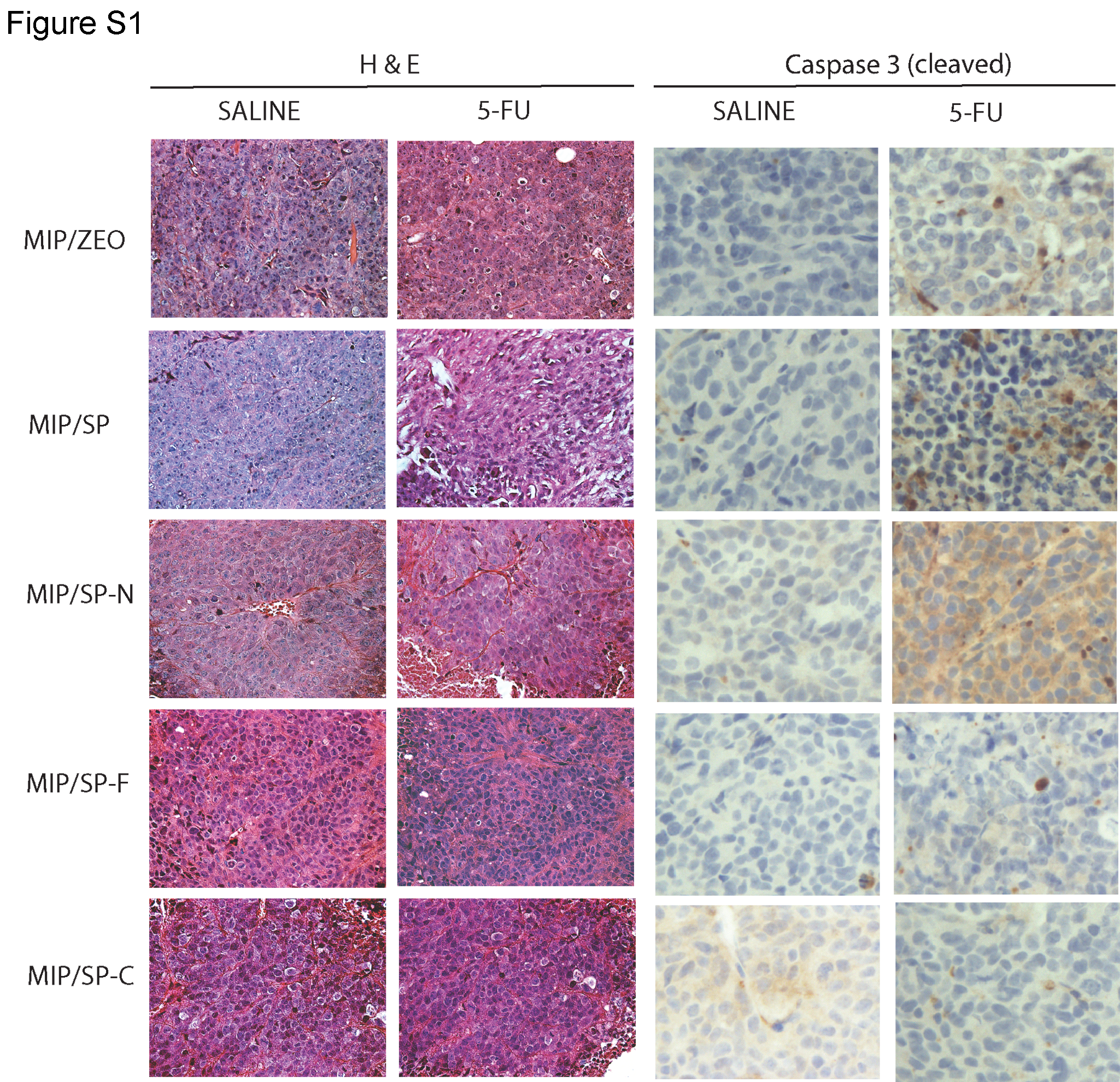

Supplement: Figure S1 — Histology of tumor xenografts. Tumor xenografts of MIP/ZEO, MIP/SP, MIP/SP-N, MIP/SP-F, and MIP/SP-C subcutaneously implanted cells were paraffin-embedded and processed and stained with Hematoxylin and Eosin (H&E), and cleaved caspase 3. (TIF) [file pone.0026390.s001.tif]

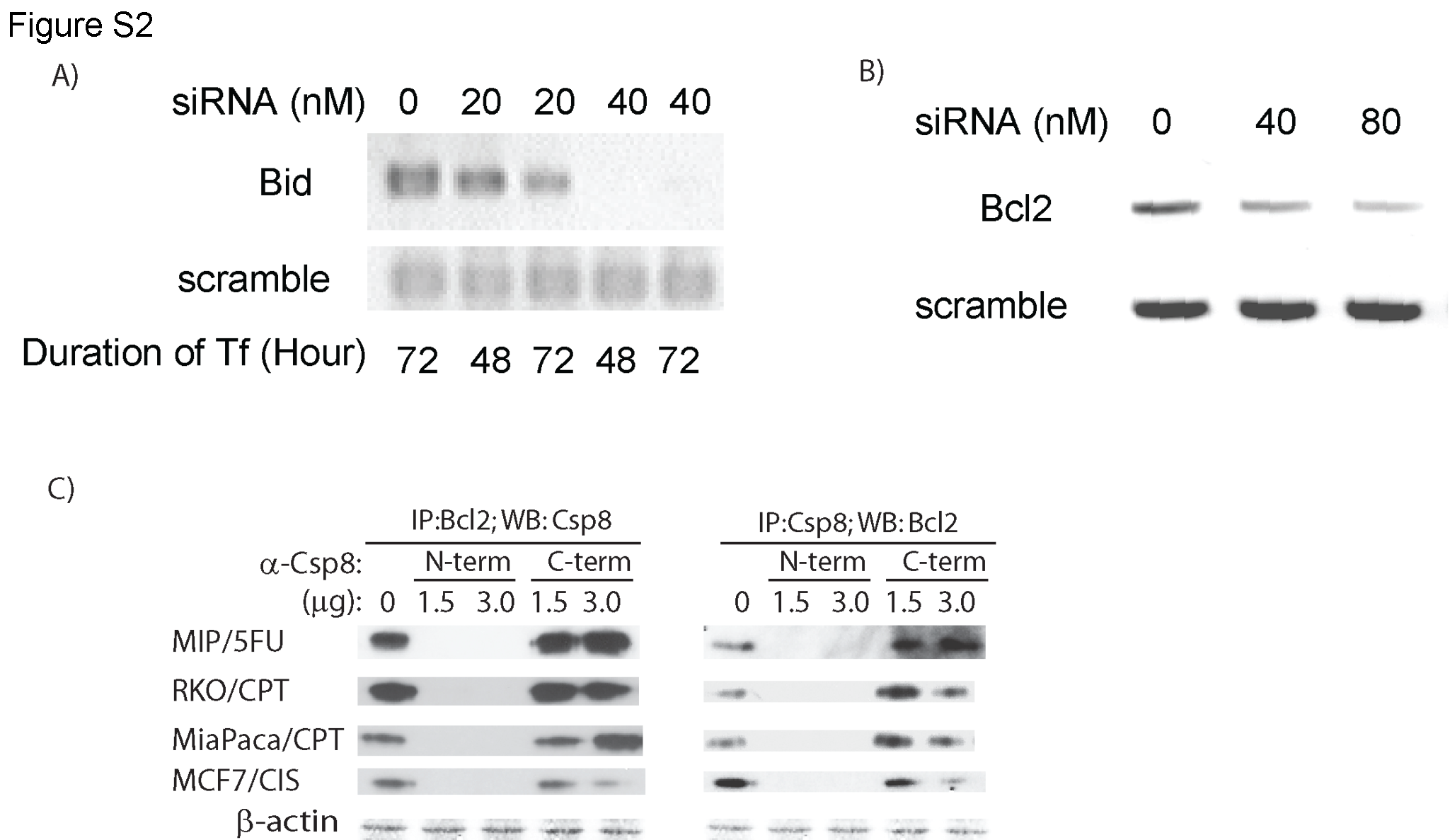

Supplement: Figure S2 — siRNA optimization for Bid and Bcl2; and interaction between Bcl2 and caspase 8 in various resistant cells. A) Bid siRNA optimization: MIP101 cells were transfected with 20–40 nM Bid siRNA (or scramble control). RNA was isolated from cells harvested after 48–72 hrs of transfection and Bid gene expression determined by RT-PCR. Optimal Bid gene expression knock-down was achieved after transfection of 40 nM of siRNA for either 48–72 hrs; B) Bcl2 siRNA optimization: MIP/5FU cells were transfected with 40–80 nM Bcl2 siRNA (or scramble control) for 72 hrs. A 40% and 60% reduction in Bcl2 gene expression was noted following transfection with 40 nM and 80 nM respectively. The optimal condition used in subsequent experiments included the transfection of cells with 80 nM of Bcl2 siRNA for 72 hrs; C) The interaction between Bcl2-caspase 8 occurs at the N-terminus of caspase 8 as cells incubated with antibodies blocking caspase 8 (N-term, 1.5–3 µg) prevented this Bcl2-caspase 8 interaction. (TIF) [file pone.0026390.s002.tif]

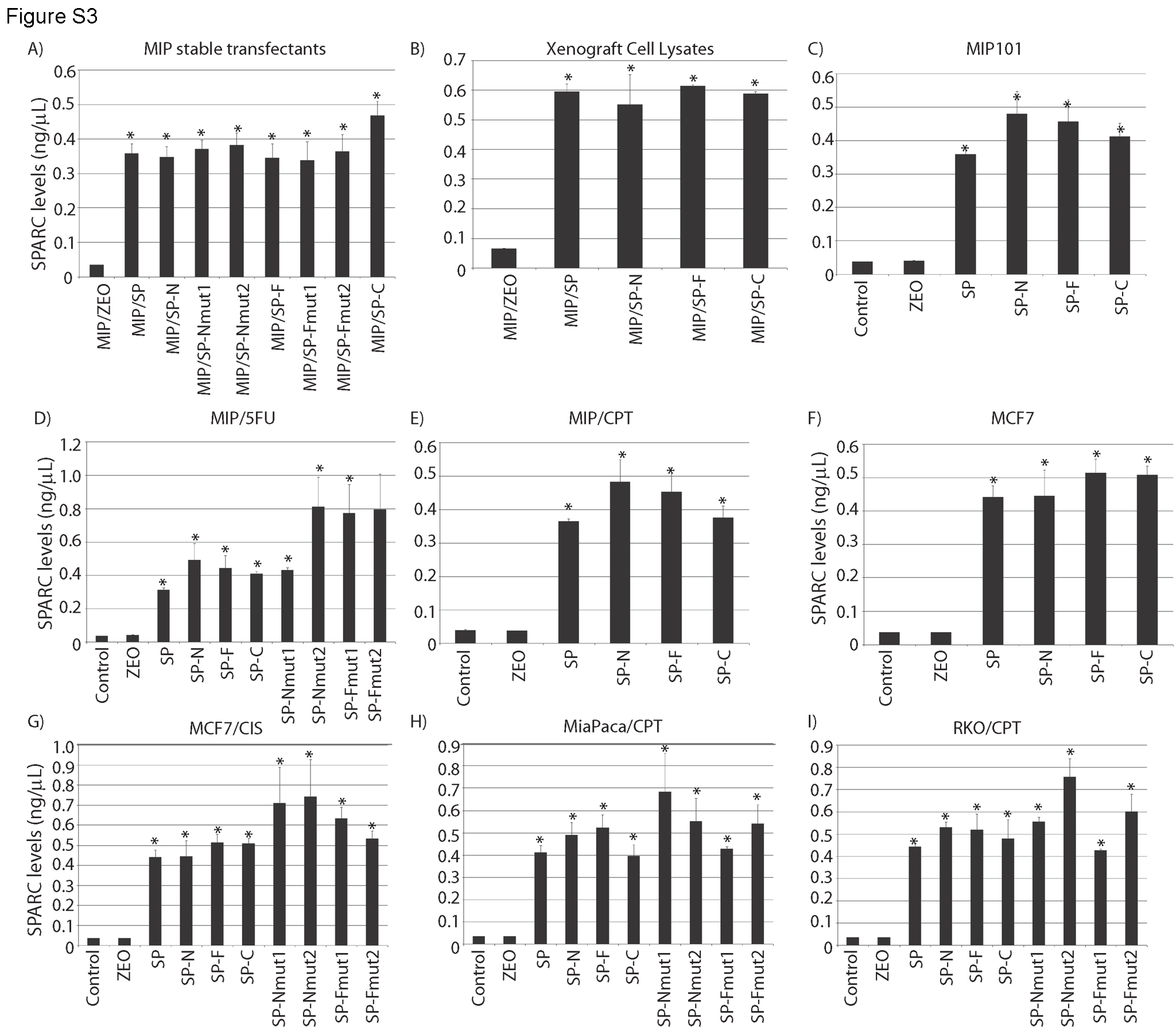

Supplement: Figure S3 — Levels of SPARC and related peptides in cells used in this study. Cell lysates from stable transfectants (in-vitro and in-vivo) or transiently transfected cells were isolated 120 hours post-transfection and levels of SPARC and SPARC-related peptide levels were assayed by ELISA. Results represent mean ± s.e. (n = 3 independent studies). Student's t-test, * statistical difference compared to control, where p<0.05. (TIF) [file pone.0026390.s003.tif]
