# Supplementary material for: A Peptide of SPARC Interferes with the Interaction between Caspase8 and Bcl2 to Resensitize Chemoresistant Tumors and Enhance Their Regression In Vivo
Source: PLoS One. 2011 Nov 1;6(11):e26390. doi: 10.1371/journal.pone.0026390 (PMC3206029; doi:10.1371/journal.pone.0026390)
Supplement: Table S1 — List of abbreviations. (DOC) [file pone.0026390.s004.doc]

**Table S1 – List of abbreviations**

| **Abbreviation** | **Complete Name** |
| --- | --- |
| 5-FU | 5-fluorouracil |
| aa | Amino acid |
| bFGF | Basic fibroblatic growth factor |
| CIS | Cisplatin |
| CPT-11 | Irinotecan |
| CRC | Colorectal cancer |
| Csp8 | Caspase 8 |
| Csp 9 | Caspase 9 |
| Csp 3 | Caspase 3 |
| DEDI | Death effector domain I of caspase 8 |
| DEDIm | Mutant of the death effector domain I of caspase 8 |
| DEDII | Death effector domain II of caspase 8 |
| DEDIIm | Mutant of the death effector domain II of caspase 8 |
| DMEM | Dulbecco’s modified eagle’s medium |
| EC | Extracellular domain of SPARC |
| ELISA | Enzyme-Linked ImmunoSorbent Assay |
| FS | Follistatin-like domain of SPARC |
| MCF7/CIS | MCF7 cells resistant to cisplatin |
| MiaPaca/CPT | MiaPaca cells resistant to irinotecan |
| MIP/5FU | MIP101 cells resistant to 5-fluorouracil |
| MIP/SP | MIP101 cells over-expressing SPARC |
| MIP/SP-C | MIP101 cells over-expressing the EC-domain of SPARC |
| MIP/SP-F | MIP101 cells over-expressing the follistatin-like domain of SPARC |
| MIP/SP-N | MIP101 cells over-expressing the N-terminal domain of SPARC |
| MIP/ZEO | MIP101 cells stably-transfected with empty vector |
| MMP | Matrix metalloproteinases |
| NT | N-terminal domain of SPARC |
| PB | Putative binding domain of caspase 8 |
| PBm | Mutations in the putative binding domain of caspase 8 |
| RKO/CPT | RKO cells resistant to irinotecan |
| rSPARC | Recombinant human SPARC |
| SAM | S-adenosylmethionine |
| siRNA | Small interfering RNA |
| SP | Full length SPARC |
| SP-C | Plasmid expressing the extracellular domain of SPARC |
| SP-F | Plasmid expressing the follistatin-like domain of SPARC |
| SP-Fmut1 | Mutant 1- plasmid expressing a mutant follistatin-like domain of SPARC |
| SP-Fmut2 | Mutant 2 - plasmid expressing a mutant follistatin-like domain of SPARC |
| SP-N | Plasmid expressing the N-terminal domain of SPARC |
| SP-Nmut1 | Mutant 1- plasmid expressing a mutant N-terminal domain of SPARC |
| SP-Nmut2 | Mutant 2 - plasmid expressing a mutant N-terminal domain of SPARC |
| SPARC | Secreted protein acidic and rich in cysteine |
