# Supplementary material for: A Peptide of SPARC Interferes with the Interaction between Caspase8 and Bcl2 to Resensitize Chemoresistant Tumors and Enhance Their Regression In Vivo
Source: PLoS One. 2011 Nov 1;6(11):e26390. doi: 10.1371/journal.pone.0026390 (PMC3206029; doi:10.1371/journal.pone.0026390)
Supplement: Table S2 — Site-directed mutagenesis primers. (DOC) [file pone.0026390.s005.doc]

**Table S2** – *Site-directed mutagenesis primers*

|  | **Sense** | **Anti-sense** |
| --- | --- | --- |
| **N-terminal domain of SPARC** | 5’ tccaccttca tcctcttaaa AATCGGTGTC CCATTTCCAT gcttctcctc 3’ | 5’ tttaagagga tgaaggtgga cctgtcctaa 3’ |
| **FS-like domain of SPARC** | 5’ gtcgttactg ttgttctgga CGTGTCGAGG AGGTTCGAGT aaacggtgtt 3' | 5’ tccagaacaa cagtaacgac gtgtggaaga 3’ |
| **DEDI domain of caspase 8** | 5’ tctttagaaa tactataacc AGGTACCATC AGGTACCGTG tagaccggag 3’ | 5’ ggttatagta tttctaaaga cgacttcagg 3’ |
| **Putative binding domain of caspase 8** | 5’ ttgac ctgtcacttc tagaAATAGC ggagttcaag 3’ | 5’ tctagaagtgacag gtcaacaagg ggtt 3’ |
| **DEDII domain of caspase 8** | 5’ gagatagtct aaagtcttct CCTAGTACAG GAGCTAACTC ccagaaaatt 3’ | 5’ agaagacttt agctatctc gtactgggac 3’ |
